# Mapping the Epileptogenic Brain Using Low-Frequency Stimulation: Two Decades of Advances and Uncertainties

**DOI:** 10.3390/jcm14061956

**Published:** 2025-03-13

**Authors:** Samuel B. Tomlinson, Michael E. Baumgartner, Timothy R. Darlington, Eric D. Marsh, Benjamin C. Kennedy

**Affiliations:** 1 Department of Neurosurgery, University of Pennsylvania, Philadelphia, PA 19104, USA; 2 Perelman School of Medicine, University of Pennsylvania, Philadelphia, PA 19104, USA; 3 Division of Child Neurology, Children’s Hospital of Philadelphia, Philadelphia, PA 19104, USA; 4 Departments of Neurology and Pediatrics, University of Pennsylvania, Philadelphia, PA 19104, USA; 5 Division of Neurosurgery, Children’s Hospital of Philadelphia, Philadelphia, PA 19104, USA

**Keywords:** cortico-cortical evoked potential, cortical stimulation, epilepsy, network, connectivity

## Abstract

Cortical stimulation is the process of delivering brief pulses of electrical current and visualizing the distributed pattern of evoked responses across the brain. Compared to high-frequency stimulation, which has long been used for seizure provocation and functional mapping, low-frequency stimulation (<1–2 Hz) is rarely incorporated into the epilepsy surgery evaluation. Increasingly, researchers have demonstrated that various cortico-cortical evoked potential (CCEP) features, including early and delayed responses, evoked high-frequency oscillations, and derived network metrics, may be useful biomarkers of tissue excitability and abnormal connectivity. Emerging evidence also highlights a potential role of CCEPs in guiding neuromodulatory therapies like responsive neurostimulation. In this review, we examine the past two decades of innovation in low-frequency stimulation as it pertains to pre-surgical evaluation. We begin with a basic overview of single-pulse electrical stimulation and CCEPs, including definitions, methodology, physiology, and traditional interpretation. We then explore the literature examining CCEPs as markers of cortical excitability, seizure onset, and network-level dysfunction. Finally, the relationship between stimulation-induced and spontaneous seizures is considered. By examining these questions, we identify both opportunities and pitfalls along the path towards integrating low-frequency stimulation into clinical practice.

## 1. Introduction

Drug-resistant epilepsy (DRE) is a debilitating condition affecting ~30% of patients with epilepsy [1]. For carefully selected patients, surgical interventions including resection, ablation, and neuromodulation can significantly reduce seizure burden and improve quality of life [2]. Achieving durable seizure control through epilepsy surgery requires precise delineation of the epileptogenic zone (EZ), i.e., the minimum expanse of brain tissue that must be surgically disrupted to prevent seizures [3]. In clinical practice, the boundaries of the EZ are inferred through an integrated evaluation of seizure semiology, radiographic lesions, and electrographic abnormalities, often captured from invasive subdural electrodes or stereoelectroencephalography (SEEG). Increasingly, researchers have refined the conceptualization of the EZ to incorporate the broader network of interconnected brain regions whose dynamic interactions provide the substrate for seizure generation and propagation [4]. Many techniques have emerged to characterize the EZ and its associated network, including structural connectivity (e.g., diffusion tensor imaging, DTI), resting-state functional connectivity, and active mapping through cortical stimulation [5,6,7,8].

Electrical stimulation allows researchers to interrogate the epileptic brain by applying repeated pulses of current and visualizing distributed patterns of evoked responses [9]. Since the pioneering work of Penfield and Jasper in the 1950s [10], high-frequency stimulation (HFS; 50–100 Hz) has been widely used to provoke seizures and map critical functional systems, including motor [11], language [12], memory [13], and limbic circuitry [14,15]. Low-frequency stimulation (LFS), often defined as the delivery of brief current pulses at a frequency < 1–2 Hz, is comparatively under-utilized in the clinical setting [16]. Beginning in the 1990s, investigations of the human limbic system demonstrated that LFS administered to the mesial temporal lobe could elicit stereotyped responses in nearby and remote regions, including contralateral mesial temporal structures [17,18]. Subsequent explorations of LFS tailored to epileptogenic mapping emerged in the early 2000s with work by Valentín and colleagues [19,20,21]. In typical LFS protocols, trains of 10–50 repeated pulses (single-pulse electrical stimulation, SPES) are administered to stimulation contacts. The evoked responses (cortico-cortical evoked potentials, CCEPs) are recorded from response electrodes and are usually averaged across repeated pulses to account for noise and response variability. Various components of the CCEP response have been investigated as markers of tissue excitability [22,23,24]. Furthermore, the presence of a CCEP response is frequently used to establish a causal relationship between the stimulation and response sites [25]. In contrast to HFS, which is considered the standard of care for seizure provocation and functional mapping at most centers, LFS is often regarded as exploratory research and is therefore less commonly incorporated into surgical decision-making.

Over the past two decades, many studies have investigated the clinical implications of LFS in epilepsy surgery planning. In this review, we explore the evolution of this field in the context of core translational questions driving progress in LFS research, including the following:

(1) Do specific response patterns differentiate epileptogenic tissues from healthy regions?
(2) How do epileptogenic regions interact with and influence the broader network of functionally connected brain regions?
(3) How do stimulation-induced seizures relate to spontaneous seizures?
(4) Can LFS guide emerging stimulation-based therapies such as responsive neurostimulation (RNS)?

## 2. Basic Principles of Low-Frequency Stimulation

LFS involves delivering brief, repeated pulses of low-frequency electrical current (SPES) through invasively implanted electrodes to elicit responses (CCEPs) from functionally connected brain regions. LFS is typically performed extra-operatively in the epilepsy monitoring unit (EMU) on patients undergoing invasive phase II monitoring for seizures.

The protocols for delivering SPES and quantifying CCEPs vary tremendously across institutions. The physical parameter space for SPES includes the stimulation frequency, current, pulse width, electrode configuration, and waveform. A typical SPES paradigm (e.g., the research protocol utilized at our institution) involves delivering trains of 10–30 repeated pulses at a frequency between 0.5 and 1 Hz, with current ranging from 1 to 10 milliamps (mA) and a pulse width between 300 and 500 microseconds (μs). This process can be repeated systematically at each adjacent pair of electrodes in the implant array. Bipolar stimulation (i.e., current delivered between two adjacent electrodes) has been shown to produce a more focused activation field compared to monopolar stimulation [26] and appears to be generally preferred in the recent literature. The shape of the electrical field generated during each pulse is determined by the arrangement of the working and reference electrodes. The stimulation waveform can significantly influence the likelihood of triggering seizures and inducing local tissue damage. Many groups favor the use of cathode-leading, biphasic, fast reversal waveforms for the favorable tradeoff between sufficient local tissue excitation and low risk of tissue injury [27]. The maximum current is usually titrated to be beneath the threshold that triggers after-discharges, which is thought to reduce the likelihood of provoking a clinical seizure [28]. Although CCEPs can be elicited intraoperatively during electrode implantation, anesthesia has been shown to influence various aspects of the CCEP response, including their consistency across repeated pulses [29,30,31]. Similarly, sleep has been shown to modulate CCEP responses [32,33]. For this reason, researchers should standardize whether stimulation is performed in an awake or asleep subject, or at the very least must document the patient’s arousal state during the stimulation session. Typically, LFS is performed after a satisfactory number of spontaneous seizures has been captured and the patient’s anti-epileptic medications have been resumed, although this is variable across centers. SPES is considered very safe when performed with appropriately selected parameters [34].

Once stimulation is completed, signal processing is required to convert raw responses into interpretable CCEPs. Systematic analysis of how CCEP responses vary with different stimulation parameters and processing pipelines is a nascent field, although several high-quality studies have emerged in recent years [27,35,36,37,38,39,40]. Common post-processing steps include filtering, de-trending, trial rejection (manual vs. automated), baseline subtraction, amplitude normalization, and grand-averaging [27]. Further nuances include scaling CCEPs based on the estimated volume conduction potential to mitigate the influence of volume conduction [41,42]. The stimulation artifact can contaminate the early response window up to 5–30 ms post-stimulation, necessitating its algorithmic removal. Frequently, post-processing is performed using customized routines developed by individual labs but may be aided by open-source packages [43]. The magnitude of the CCEP response can be reported as a maximum amplitude within a pre-specified time window, or as the integrated area or root-mean-square (RMS) amplitude [44]. Many groups also calculate CCEP latencies, interpreting transmission velocity as an additional measure of connection strength between the stimulation and response sites.

**Figure 1:**
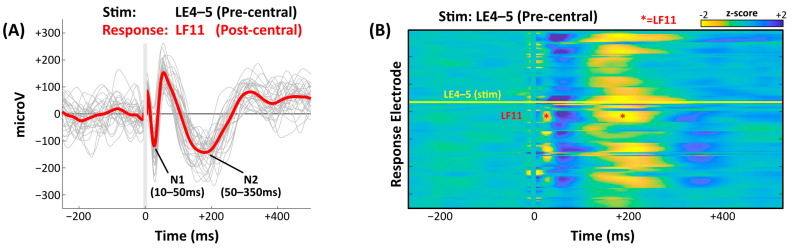
**Illustrative CCEPs evoked by SPES.** (**A**) Repeated pulses (*n* = 30) were delivered through contact pair LE4-5 (pre-central gyrus) at a frequency of 1 Hz. Individual trial (gray) and grand-averaged (red) CCEPs are demonstrated at the response contact (LF11, post-central gyrus). Canonical N1 and N2 components are evident in both the individual and grand-averaged waveforms. The vertical gray line represents the −5:+5 ms peri-stimulus interval, which was excluded. (**B**) Distribution of grand-averaged CCEPs across response electrodes (*n* = 130) following stimulation at LE4-5 (yellow line). Red asterisks denote the N1 and N2 responses for the illustrative response contact, LF11. In this panel, CCEPs were z-score-normalized prior to grand-averaging.

Several components of the trial-averaged CCEP waveform have been described, including the canonical N1 and N2 components (Figure 1). The N1 constitutes a negative waveform deflection occurring between 10 and 50 ms post-stimulation, likely reflecting local excitation induced by mono- or oligo-synaptic projections from the stimulation site [45]. The N2 (50–350 ms) is thought to reflect the complex integration of multiple processes including poly-synaptic input, cortico-subcortico-cortical projections, and post-excitatory inhibition [45,46]. Conventionally, N1 and N2 comprise the first and second negative waveform deflections following stimulation, but the polarity of the response waveform is dictated by the dipole orientation and may therefore vary across response sites, especially in the three-dimensional recording fields of SEEG [24]. Previous studies have demonstrated that CCEP amplitudes generally decrease with increasing physical distance from the stimulation site [23,38]. Furthermore, regional relationships are often directional in that eliciting a robust response from site B after stimulating A does not guarantee reciprocal activation of A when stimulating B [47,48,49]. In addition to anti-seizure medications and anesthesia, other factors thought to influence CCEP responses (amplitude and/or velocity) include age (presumably related to maturation of axonal myelination) [50] and endogenous neural rhythms such as local spontaneous alpha/beta oscillations [51].

## 3. Do Specific Response Patterns Differentiate Epileptogenic Tissues from Healthy Regions?

The most common translational question asked in LFS studies is whether CCEPs aid in defining the EZ [22,23]. Before summarizing these studies, two important caveats must be issued. First, the absence of universal standards dictating how CCEPs should be elicited and reported hinders generalization across studies. Second, most studies are small, single-institution series examined retrospectively. To date, no large-scale, multi-institutional, prospective studies have established a conclusive link between CCEP response patterns, SOZ localization, and resection.

### 3.1. Early Response, Late Response, and CCEP Amplitude

The first series of studies examining CCEPs in relation to epileptogenicity emerged from Kings College of London in the early 2000s [19,20,21,28,52,53,54,55]. This group formalized the distinction between what they deemed the ‘early response’ (ER; <100 ms, widely present, presumably physiologic) and the ‘delayed response’ (DR; <1 s post-stimulation, infrequently elicited, electrographically resembling an interictal spike, presumably pathologic). In these studies, ERs were observed ubiquitously across both normal and epileptogenic brain regions, whereas DRs were specific to the SOZ. Resection of brain regions exhibiting DRs was associated with favorable surgical outcomes in both adult [21] and pediatric [28] cohorts. DRs were therefore proposed as an objective marker of the EZ. While Valentín and colleagues considered ERs to primarily reflect physiologic functional connectivity between the stimulation and response sites, subsequent work by Mouthaan et al. [56] reported that electrodes with a higher frequency of suprathreshold ERs tended to localize to the SOZ, and that when stimulating the SOZ, ERs were preferentially elicited in regions involved in seizure propagation. This suggests that ERs may index regional differences in excitability and connectivity with the SOZ.

Several groups have examined the relationship between peak CCEP amplitudes and epileptogenicity. One study of 11 patients demonstrated that stimulation of SOZ contacts elicited larger N1 responses in the ictal propagation zone compared to non-involved control regions [57]. Lega et al. [58] similarly reported increased CCEP RMS (20–400 ms) in the early seizure propagation zone compared to the late recruitment area following stimulation of SOZ contacts. Tousseyn et al. [59] performed an integrated analysis of CCEPs and ictal single-photon emission computed tomography (SPECT), finding significantly larger evoked responses in hyperperfused compared to baseline-perfused regions. In a small series of eight patients undergoing systematic 1 Hz stimulation for CCEPs, Iwasaki et al. [60] identified accentuated N1 amplitudes (5–80 ms) within the SOZ compared to non-SOZ regions in seven out of eight patients (87.5%). Zhang et al. [61] corroborated this observation in their study of 15 patients subjected to 1 Hz stimulation, finding that the CCEP RMS (7–300 ms) was higher in SOZ than in control regions, particularly among patients with a repetitive spiking electrographic seizure onset pattern. Enatsu et al. [62] provided further evidence for a relationship with seizure onset pattern, finding that CCEP responses (0–200 ms) were larger and more spatially distributed when stimulation was applied to SOZ versus non-SOZ contacts, especially in patients with repetitive spiking onset compared to paroxysmal fast onset.

Intriguingly, responses across varying stimulation intensities may provide further information about local excitability. Using a stimulation titration protocol, Hays et al. [63] found that the increase in N1 amplitudes with gradual up-titration of stimulation intensity was greater in SOZ compared to non-SOZ regions. This effect was most dramatic when stimulation was administered to the SOZ. Increasingly, application of sophisticated techniques such as deep learning [64,65], state-space models [66], and adaptive transfer functions [67] have been used to enhance the accuracy SOZ localization from CCEPs. These studies, which require validation in prospective trials, suggest that CCEP response patterns may index variations in local tissue excitability relevant for EZ localization.

### 3.2. Evoked High-Frequency Oscillations

Further exploration of evoked responses in the time–frequency domain reveal that stimulation can induce high-frequency oscillations (HFOs). HFOs occur spontaneously in patients with epilepsy and are typically divided into ripples (80–250 Hz) and fast ripples (FRs; 250–500 Hz) [68]. Spontaneous HFOs are thought to arise when excitable neural populations become transiently and pathologically synchronized, resulting in a rhythmic burst of discharges [69]. Spontaneous HFOs have been observed both in isolation and in association with interictal spikes, particularly within the SOZ [70]. Resection of regions exhibiting spontaneous HFOs (especially FRs) has been associated with better surgical outcomes [71].

Evoked HFOs are presumed to reflect similar local tissue excitability and have therefore been investigated as a potential EZ biomarker [53]. Van Klooster et al. [72] provided the initial characterization of HFOs induced by SPES. In a subsequent study of 10 patients, SPES triggered FRs in the SOZ even in some patients for whom spontaneous FRs had not been captured [73]. Evoked HFOs occur more readily in areas with a lower stimulation threshold for triggering after-discharges and habitual seizures [74]. Donos et al. [75] examined the co-occurrence of HFOs and DRs in 16 patients, finding that regions frequently exhibited both types of responses, especially within the SOZ. Kobayashi et al. [76] demonstrated that stimulation-induced increases in high-frequency spectral power during the N1 window were greater within SOZ compared to non-SOZ regions, especially in the mesial temporal lobe. Intriguingly, several studies have shown that after an initial post-stimulation window of increased high-frequency activity, many regions exhibit a delayed high-frequency suppression (0.4–1 s after stimulation), particularly within the SOZ or following SPES in the SOZ [77,78]. This delayed inhibitory response is postulated to reflect an inhibitory surrounding that functionally isolates the SOZ from the broader epileptogenic network, although this explanation requires further scrutiny. Taken together, these studies provide preliminary support for the hypothesis that patterns of evoked HFOs, much like spontaneous HFOs, can aid in identifying regions of increased epileptogenicity.

### 3.3. CCEPs and Interictal Spikes

Interictal spikes are an electrographic hallmark of epilepsy thought to arise from the paroxysmal, synchronous depolarization of local cortical ensembles. Compared to HFOs, interictal spikes are generally considered less specific markers of the EZ [79], and the relationship between spikes and seizures has been controversial for decades [80,81]. The spatial distribution of interictal spikes tends to exceed the areas involved in seizure onset and early propagation, suggesting that spikes can arise independently or spread to regions beyond the presumed EZ [82].

To date, few studies have examined the relationship between CCEPs and interictal spikes. One study by Alarcon et al. [52] probed this relationship using microelectrode recordings to compare single-neuron firing patterns during both spontaneous interictal spikes and CCEPs. They described four distinct firing patterns (burst-only, suppression-only, burst–suppression, and no change) which were remarkably consistent between CCEPs and spikes, suggesting that these distinct phenomena share similar physiologic mechanisms. Nayak et al. [83] formulated their experimental question as follows: does stimulation evoke a response pattern that is topographically similar to interictal spike discharges? In a cohort of 36 patients undergoing SPES, they found that 28/36 patients (77.8%) exhibited a distribution of early CCEP responses (<100 ms) that matched at least one spontaneous interictal spike pattern, suggesting that specific stimulation locations can activate networks involved in spike generation. Furthermore, among the subset of patients exhibiting DRs, all DRs resembled at least one interictal spike pattern and always localized to the SOZ, suggesting that the spatiotemporal correlation between DRs and interictal spikes is a marker of epileptogenic tissue. Given that patients frequently exhibit multiple independent or semi-independent interictal spike patterns, this study raises the intriguing possibility of stratifying distinct spike patterns by testing whether they can be reproduced through active stimulation. Further work validating this concept is needed before determining its clinical utility.

### 3.4. CCEP Variability

CCEP responses are highly variable even when stimulation is performed using the same experimental protocols [84]. This variability can be appreciated between individual pulses (i.e., trials) within a stimulation train. Traditionally, researchers interpret this variability as noise and use strategies such as grand-averaging across trials to mitigate its impact. Recently, interest has emerged in leveraging inter-trial variability as a potential source of information about epileptogenicity. Feys et al. [85] quantified the inter-trial standard deviation of CCEP amplitudes and latencies, finding higher levels of both within the EZ compared to control regions. Furthermore, they found that CCEP variability decreased in patients with higher seizure frequency, suggesting that seizures may reinforce epileptogenic networks and reduce their variability over time through mechanisms like synaptic remodeling. Cornblath et al. [86] similarly reported increased inter-trial variability of N1 and N2 amplitudes in SOZ regions, in addition to regions with higher interictal spike rates. They further demonstrated that CCEPs exhibit rare monotonic trends (i.e., gradual increase or decrease in response amplitudes across trials), and that stimulating areas with higher interictal spike rates resulted in more positive monotonic trends of N1 and N2. These two studies challenge the notion that CCEP variability should be discounted and may in fact provide valuable insights regarding tissue excitability and connectivity with the SOZ.

## 4. How Do Epileptogenic Regions Interact with and Influence the Broader Network of Functionally Connected Brain Regions?

Increasingly, the epileptic brain is understood as a distributed network of functionally and structurally connected regions [87]. This view has largely supplanted the notion of a discrete EZ that can be isolated and removed from the surrounding “healthy” brain. Rather, emphasis is placed on characterizing dynamic patterns of network connectivity that enable seizure onset and propagation. Within this framework, brain regions are represented as nodes, connections between nodes (structural, functional, and effective) are represented as edges, and the topology of the network (i.e., the distribution of edges between nodes) can be summarized using mathematical concepts from network science [88].

### 4.1. Effective Connectivity Measures and SOZ Localization

CCEPs offer direct evidence of effective connectivity between stimulation and response nodes [49]. Previous studies have utilized CCEPs to relate specific connectivity patterns to variables such as histopathology [89,90], surgical outcome, and SOZ (reviewed in [46]). Van Bloojis et al. [91] utilized CCEPs to construct effective networks in 21 patients who underwent 0.2 Hz stimulation, finding that SOZ nodes had dense connectivity with other SOZ nodes but relatively sparse connectivity (particularly afferent/incoming connectivity) with the non-SOZ surround. This was particularly apparent in patients rendered seizure-free by resection of the SOZ. Guo et al. [92] similarly demonstrated that SOZ nodes were richly interconnected, especially in seizure-free patients. Boido et al. [93] utilized a network-based methodology in their study of 12 patients subjected to 1 Hz stimulation, finding that SOZ nodes had more bidirectional connections for early CCEP responses (<60 ms) compared to non-SOZ regions.

Several groups have identified node-level characteristics that distinguish SOZ from non-SOZ nodes. For example, Yan et al. [94] introduced the “connectivity index” as a measure of each node’s incoming and outgoing connectivity strength, finding significantly elevated values for SOZ compared to non-SOZ nodes. Another group constructed effective networks based on stimulation-induced spectral perturbations in 27 patients undergoing 0.2–1 Hz stimulation, finding that measures of node centrality accurately classified the SOZ [95]. Hays et al. [96] utilized a graph-theoretic approach to characterize CCEP connectivity in the epileptogenic mesial temporal lobe (MTL), finding greater outgoing connectivity from the MTL in patients with temporal-onset seizures compared to those with seizures arising elsewhere in the brain. Although each study suffers from limitations (e.g., small sample size, retrospective design), in the aggregate, they suggest that effective connectivity maps derived from CCEPs may help identify the SOZ nodes and describe their functional integration with surrounding structures.

### 4.2. CCEPs and Other Connectivity Measures

CCEPs represent a measure of effective connectivity wherein a causal relationship is inferred between the stimulation and response sites. Many other techniques for visualizing epileptic network connectivity have emerged, leading researchers to question how CCEP connectivity relates to these other measures. Studies assessing structural white-matter connections with DTI have demonstrated extensive overlap between the structural connectome and effective networks derived from CCEPs, supporting the intuitive notion that white-matter projections provide an anatomic substrate for CCEPs [8,97]. Functional connectivity networks computed during the resting state (e.g., waveform cross-correlation or spectral coherence) also correlate with CCEPs [98], although the correlation strength appears to be fairly modest. Indeed, in the most comprehensive study to date, Crocker et al. [25] systematically compared resting-state functional connectivity (cross-correlation, coherence), effective connectivity (Granger causality, phase lag index), structural connectivity (DTI), and evoked connectivity (CCEPs). They found that local and remote CCEP responses correlated differentially with these various connectivity measures. Local CCEPs (<30 mm) were most strongly correlated with resting-state functional connectivity (especially 10–20 Hz coherence networks), whereas long-range CCEPs (>30 mm) correlated more strongly with the DTI network. These studies represent important steps towards understanding the structural and functional scaffold constraining CCEP responses.

## 5. How Do Stimulation-Induced Seizures Relate to Spontaneous Seizures?

**Figure 2:**
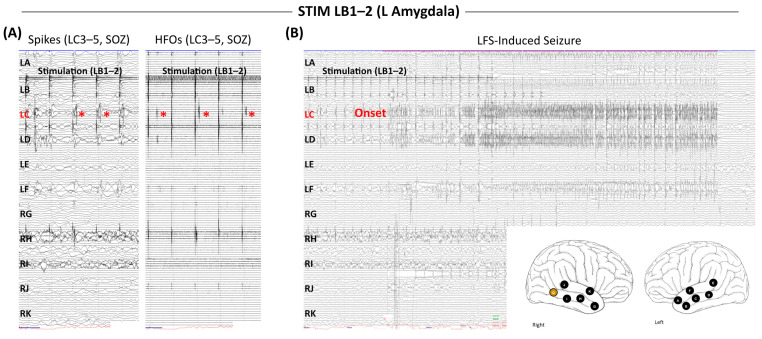
**Stimulation-induced spikes, high-frequency oscillations (HFOs), and seizures**. This representative patient underwent bi-temporal SEEG exploration. Spontaneous seizure onset zone (SOZ) was noted in the left hippocampal head (LC3-5). (**A**) SPES of the left amygdala (LB1-2) triggered discrete epileptiform spikes (left) and HFOs (right; 80 Hz high-pass filter) in LC3-5 (red asterisks). (**B**) Stimulation of LB1-2 provoked a seizure that was electro-clinically concordant with the patient’s habitual seizures, with earliest electrographic change at LC3-5.

The bulk of the literature relating stimulation-induced seizures to spontaneous seizures involves the use of HFS [99,100,101,102]. Although most LFS protocols are not designed to provoke seizures, they do occasionally occur (Figure 2), and it is often unclear whether these LFS-induced seizures provide reliable insights about EZ localization. In an early study of this topic, Munari et al. [103] demonstrated that LFS-induced seizures were much less common than HFS-induced seizures but tended to recapitulate the patient’s habitual seizures, whereas HFS-induced seizures were often electro-clinically discordant. This general pattern has been replicated in subsequent studies. Oderiz et al. [104] conducted a large study of 103 patients undergoing HFS (50 Hz) and LFS (1 Hz), observing stimulation-induced seizures in 18.2% of LFS patients compared to 54.9% of HFS patients. They further observed a relationship between surgical outcome and resection of stimulation sites that triggered seizures. Manokaran et al. [105] conducted the only study specific to the pediatric population, finding that systematic 1 Hz stimulation provoked seizures in 4/14 patients (28.6%), and that all induced seizures were concordant with the patient’s typical semiology. The small sample size of this study precluded assessment of resection and surgical outcome. Sivaraju et al. [106] designed a systematic LFS (1 Hz) protocol with gradual up-titration of stimulation current at each stimulation site (1, 5, and 10 mA). Using this approach, 23/41 patients (56.1%) undergoing 1 Hz stimulation experienced a stimulation-induced seizure, indicating that LFS can induce seizures at a rate comparable to HFS when current is titrated to a relatively high level at each site. As with prior studies, this group found that most LFS-induced seizures were concordant with habitual seizures (19/23, 82.6%). They further found that out of 11 patients rendered seizure-free by resection, all 11 had habitual seizures induced by LFS and had complete resection of those stimulation sites. These researchers and others have posited that LFS-induced seizures may provide confirmatory evidence for the accuracy of the localization hypothesis; that is, that the electrode implant has appropriately sampled the EZ, and that the true seizure generators have been identified. Further, compared to HFS, LFS-induced seizures appear to more reliably capture the patient’s spontaneous seizure circuitry, suggesting that these seizures can be more confidently integrated into the surgical evaluation.

## 6. Can LFS Guide Emerging Stimulation-Based Therapies?

Increased adoption of neuromodulatory techniques like RNS has expanded the scope of epilepsy surgery and raised many new questions about surgical planning. Key among these challenges is how to identify favorable RNS candidates and determine the optimal stimulation targets [107]. Several important studies have examined these questions in relation to resting-state connectivity (for comprehensive discussion, see [108]). Fan et al. [109] used non-invasive magnetoencephalography (MEG) to characterize pre-RNS global network connectivity in 31 patients, finding that connectivity in the alpha and beta frequency ranges could distinguish favorable RNS responders (>50% seizure reduction at >2 years) from unfavorable responders with high classification accuracy. Scheid et al. [110] examined pre-RNS functional connectivity profiles from invasive EEG recordings in 30 patients. They found that a derived network measure of ictal synchronizability in the high-gamma frequency range reliably differentiated eventual RNS responders from non-responders. One study has specifically investigated the role of CCEPs in guiding RNS therapy. Kobayashi et al. [111] assessed the utility of CCEPs to inform RNS target selection in 12 patients who underwent 1 Hz stimulation for CCEPs prior to RNS. They found that stimulating nodes with greater incoming CCEP connectivity correlated with greater eventual seizure reduction from RNS. This study provides the first evidence that CCEPs may have a role in selecting optimal RNS targets. This exciting development should motivate many more studies exploring LFS as a technique to enhance RNS planning.

## 7. Conclusions

Low-frequency stimulation is a promising methodology allowing researchers to characterize distributed patterns of effective connectivity between stimulation and response regions. The spatial topology, amplitude, variability, and high-frequency spectral perturbations associated with CCEPs likely differ within the epileptogenic zone, providing insights into local tissue excitability and connectivity. Stimulation of seizures with LFS is less common than with HFS but usually recapitulates the patient’s typical seizures and may help confirm the accuracy of the localization hypothesis. Finally, preliminary evidence suggests that LFS may have a role in guiding chronic neuromodulatory approaches like RNS. Continued exploration of LFS in epilepsy surgery, especially in the form of large-scale prospective validation trials, is needed to justify confident assimilation into the routine clinical work-flow.

## Abbreviations

The following abbreviations are used in this manuscript:

CCEPs	Cortico-cortical evoked potentials
DRE	Drug-resistant epilepsy
DR	Delayed response
DTI	Diffusion tensor imaging
EMU	Epilepsy monitoring unit
ER	Early response
EZ	Epileptogenic zone
FRs	Fast ripples
HFOs	High-frequency oscillations
HFS	High-frequency stimulation
LFS	Low-frequency stimulation
MEG	Magnetoencephalography
MTL	Mesial temporal lobe
RMS	Root mean square
RNS	Responsive neurostimulation
SEEG	Stereoelectroencephalography
SOZ	Seizure onset zone
SPES	Single-pulse electrical stimulation
SPECT	Single-photon emission computed tomography
